# Supplementary material for: Acquired IFNγ resistance impairs anti-tumor immunity and gives rise to T-cell-resistant melanoma lesions
Source: Nat Commun. 2017 May 31;8:15440. doi: 10.1038/ncomms15440 (PMC5460020; doi:10.1038/ncomms15440)
Supplement: Supplementary Information — Supplementary Figures and Supplementary Tables [file ncomms15440-s1.pdf]

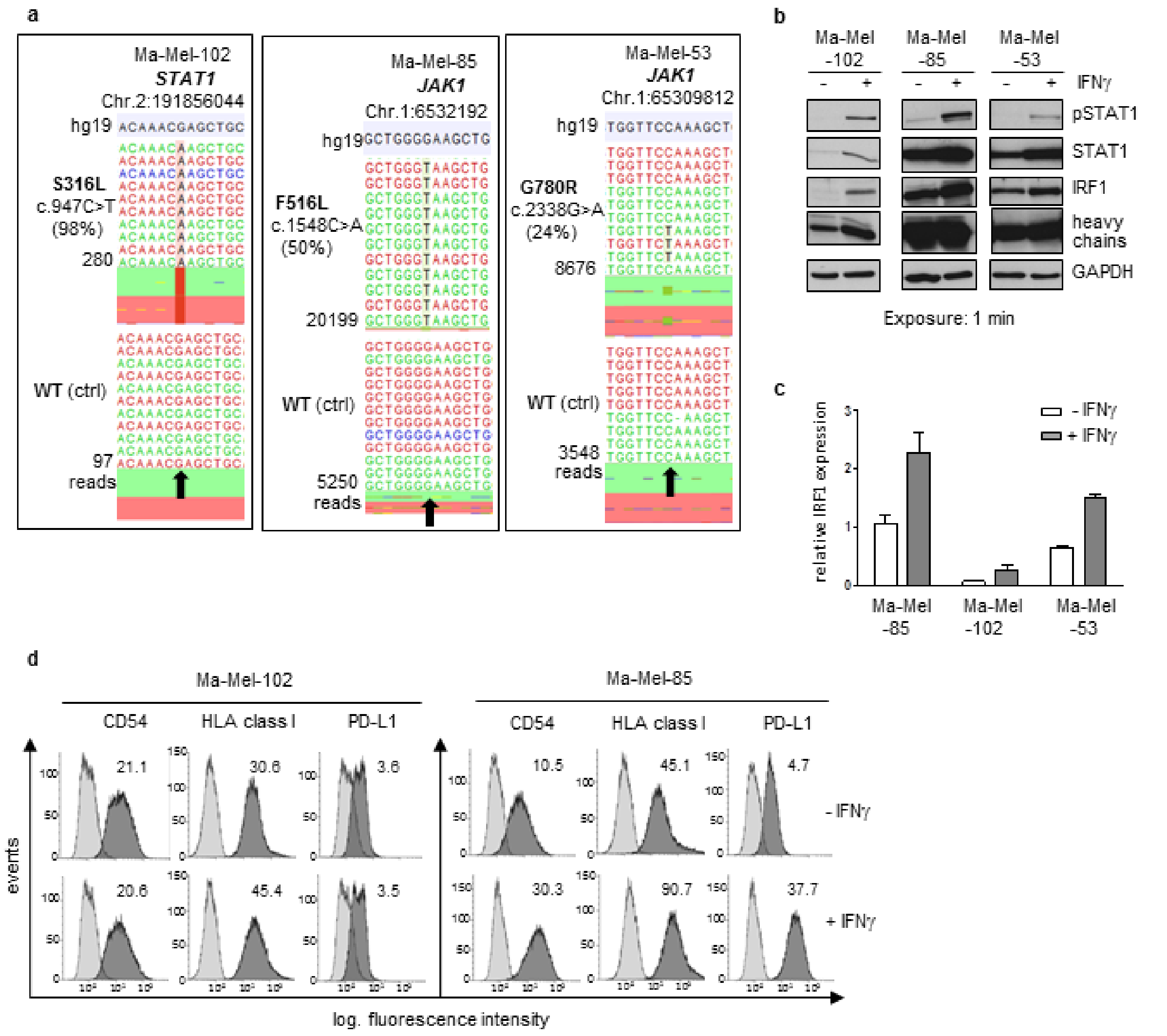

Supplementary Figure 1| *STAT1* mutation impairs IFNγ signaling. (**a**) Mutations defined by targeted sequencing on DNA from Ma-Mel-102, Ma-Mel-85 and Ma-Mel-53 cell lines and autologous blood cells as wild-type (WT) control (ctrl). Plots of aligned sequencing reads in the locations where *STAT1* c.947C>T, p.S316L, *JAK1* c.1548C>A, p.F516L and *JAK1* c.2338G>A, p.G780R mutations were identified. WT sequences shown on the bottom, arrows highlight mutation sites. Number of sequencing reads notated on the left; %, frequency of mutations in reads. (**b**) Lysates from IFNγ-treated (48 h) melanoma cells analyzed in parallel by Western blot for expression of pSTAT1, STAT1, IRF1 and HLA class I heavy chains; GAPDH, loading control. Representative data from n=2 independent experiments. (**c**) Quantification of IRF1 expression among all samples using the image processing program imageJ. IRF1 expression values were normalized to endogenous GAPDH. Mean values and SEM (error bars) obtained from n=2 independent experiments. (**d**) Surface expression of CD54, HLA class I and PD-L1 on IFNγ-treated (48 h) melanoma cells, measured by flow cytometry. Representative data from n=3 independent experiments.

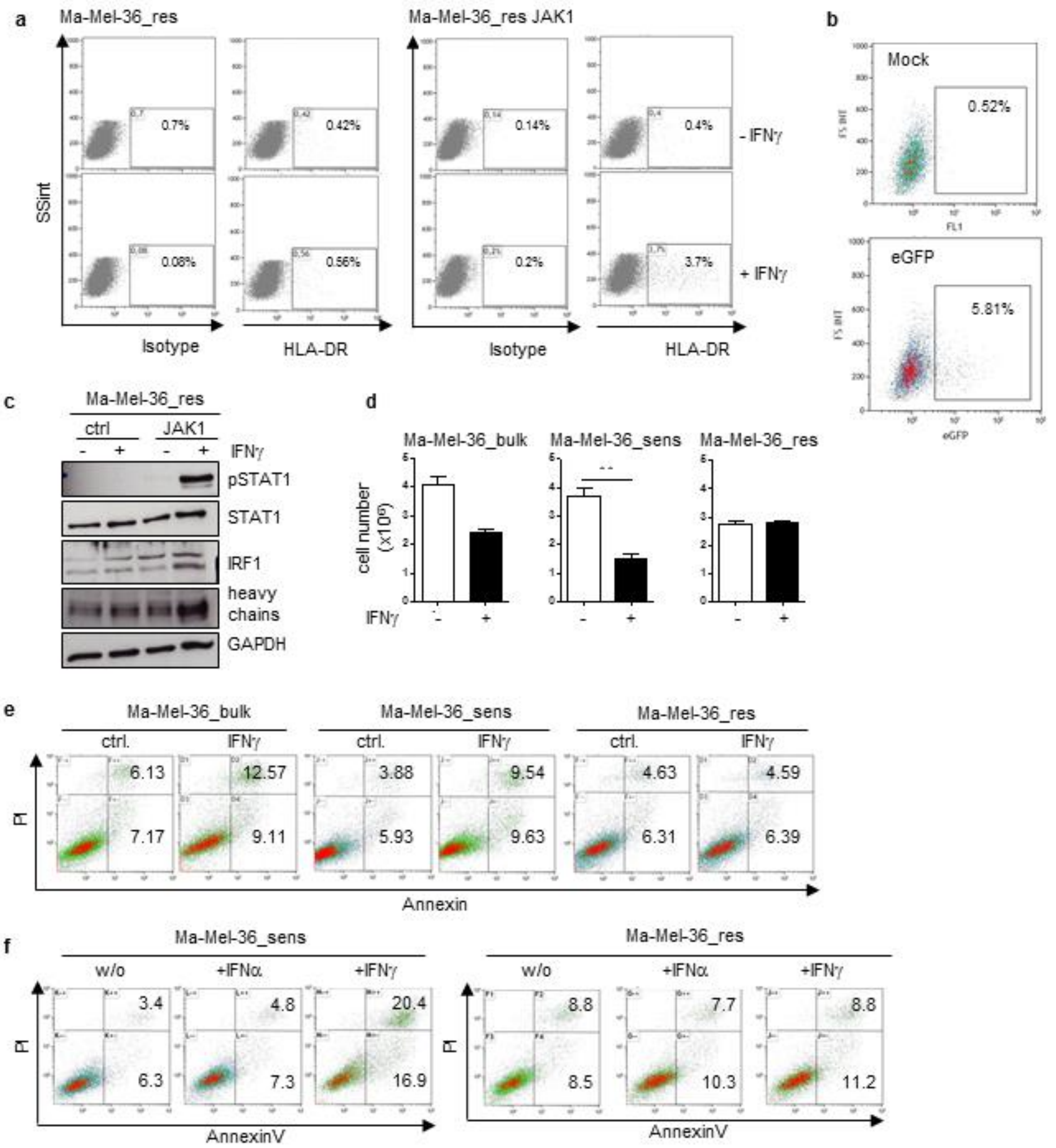

Supplementary Figure 2| JAK1 re-expression in Ma-Mel-36_res cells restores IFNγ signaling. (**a**) Melanoma cells transiently transfected with a *JAK1* expression plasmid and treated with IFNγ (500 U/ml) for 48 h. Non-transfected cells served as control. Surface expression of HLA-DR measured by flow cytometry. Representative data from n=2 independent experiments. (**b**) Transfection efficiency of Ma-Mel-36_res cells determined by eGFP plasmid transfection. Representative data from n=2 independent experiments. (**c**) Lysates from IFNγ-treated (48 h) *JAK1*-transfected and control Ma-Mel-36_res cells analyzed by Western blot for expression of indicated proteins; GAPDH, loading control. (**d**) Numbers of vital Ma-Mel-36_bulk, Ma-Mel-36_sens and Ma-Mel-36_res cells after IFNγ treatment (7 d). Only statistical significant differences defined by paired Student's t-test are indicated, **p<0.002. Mean values and SEM (error bars) obtained from n=3 independent experiments. (**e**) IFNγ-induced (7 d) apoptosis in melanoma cells determined by AnnexinV/PI staining in flow cytometry. (**f**) Comparison of IFNα and IFNγ treatment (7 d) for apoptosis induction in melanoma cells by AnnexinV/PI staining. (**e, f**) Representative dot plots from three (**e**) and two (**f**) independent experiments, indicating early (AnnexinV+/PI-) and late apoptotic (AnnexinV+/PI+) cells.

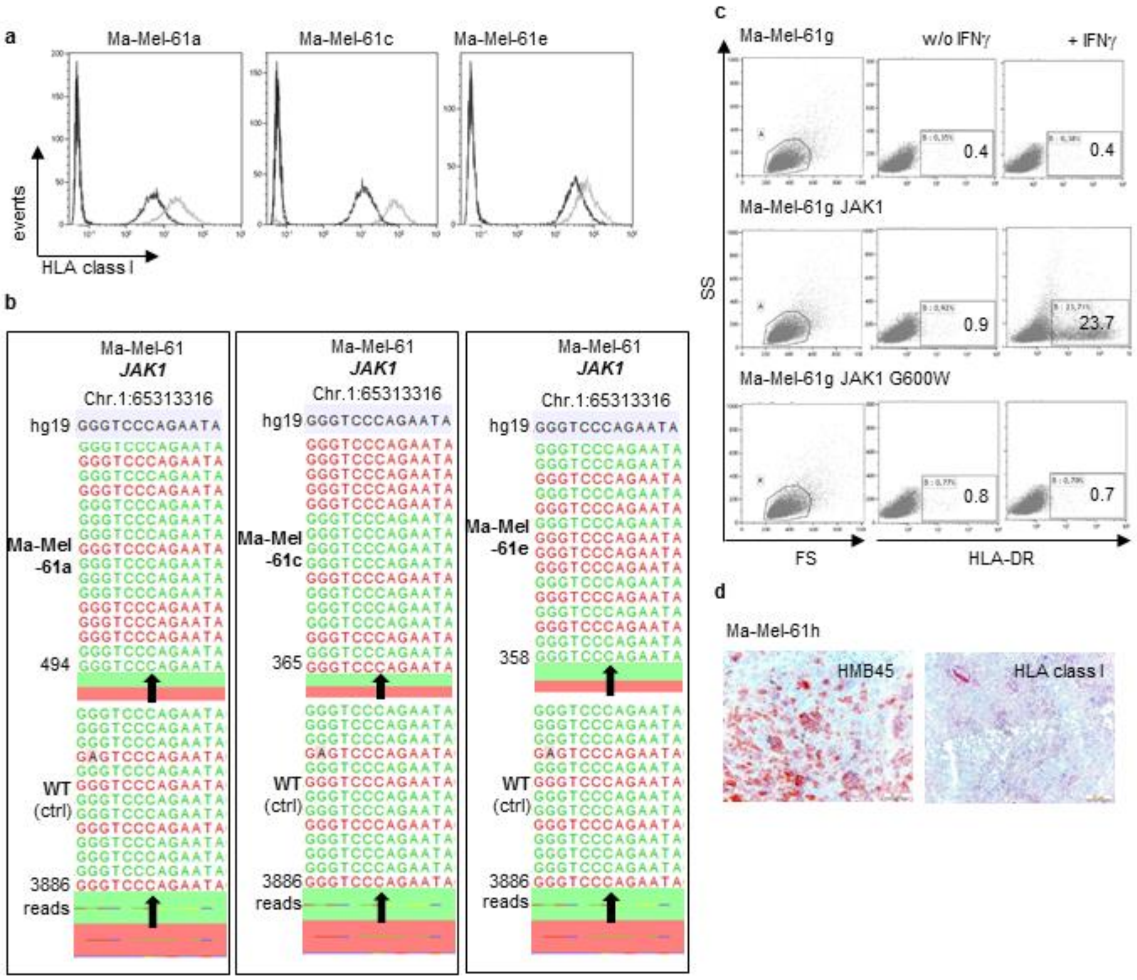

Supplementary Figure 3| JAK1 re-expression in Ma-Mel-61g cells restores IFNγ signaling. (**a**) Surface expression of HLA class I on Ma-Mel-61a, Ma-Mel-61c and Ma-Mel-61e cells treated with IFNγ (500 U/ml) for 48 h, measured by flow cytometry. Representative data from n=2 independent experiments. (**b**) No *JAK1* mutation in Ma-Mel-61a, Ma-Mel-61c and Ma-Mel-61e cells. Targeted *JAK1* sequencing on DNA from Ma-Mel-61a, Ma-Mel-61c and Ma-Mel-61e cells and autologous blood cells as wild-type (WT) control (ctrl). Plots of aligned sequencing reads, WT sequences shown on the bottom, arrows highlight the mutation site *JAK1* c.1798G>T, p.G600W detected in Ma-Mel-61g cells. Number of sequencing reads notated on the left. (**c**) Ma-Mel-61g cells transiently transfected with expression plasmids encoding JAK1 or JAK1 G600W treated with IFNγ (500 U/ml) for 48 h. Non-transfected cells served as control. Surface expression of HLA-DR measured by flow cytometry. Representative data from n=3 independent experiments. (**d**) Immunohistochemical staining of serial cryostat tissue sections from metastasis Ma-Mel-61h for melanoma marker HMB45 and HLA class I.

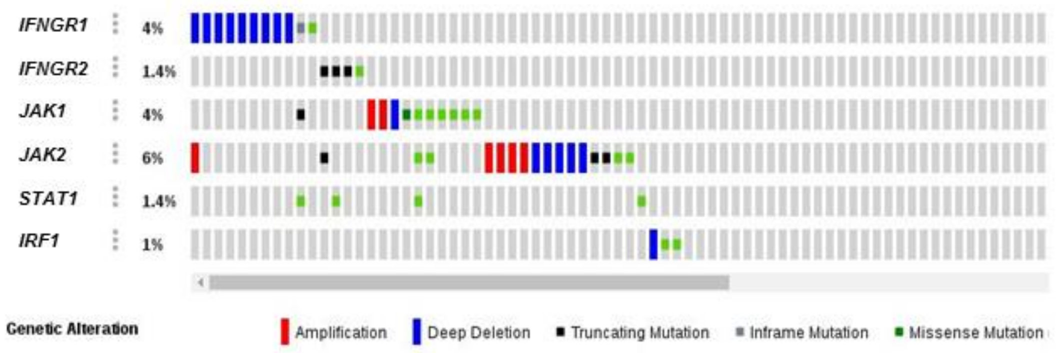

Supplementary Figure 4| Mutation co-occurrence in melanoma tumor samples. TCGA melanoma samples (n=287) with sequencing and CNV data were analyzed for co-occurence of genetic alterations. *JAK1* and *STAT1* mutations show a tendency for co-occurrence (p=0.008, log OR>3), although only 2 out of 4 *STAT1* mutations co-occur with 2 out of 11 *JAK1* mutations.

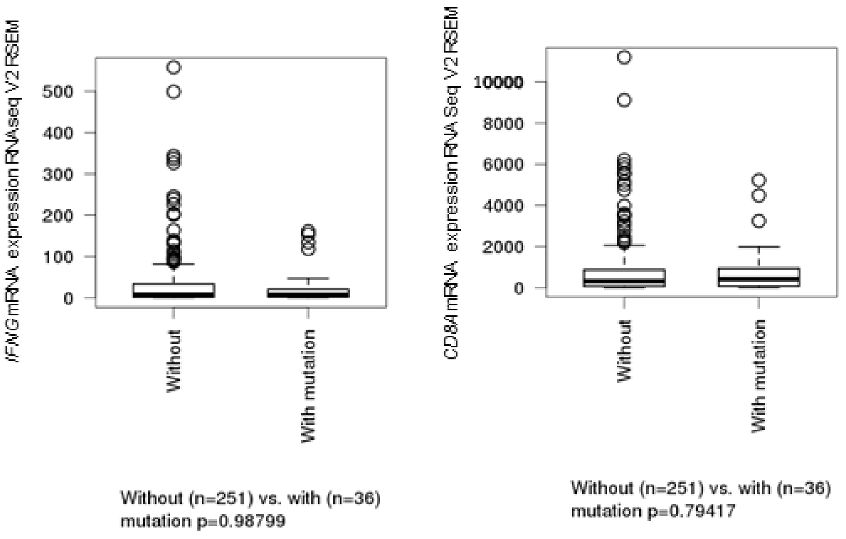

Supplementary Figure 5| *IFNG and CD8A* mRNA expression in tumors with and without mutations. TCGA melanoma samples (n=287) with available data for SNV/Indels and CNV (homdels) were studied for *IFNG* and *CD8A* mRNA expression. Two-sided Wilcoxon rank test revealed no differences in specific mRNA expression levels between tumor with and without mutations.

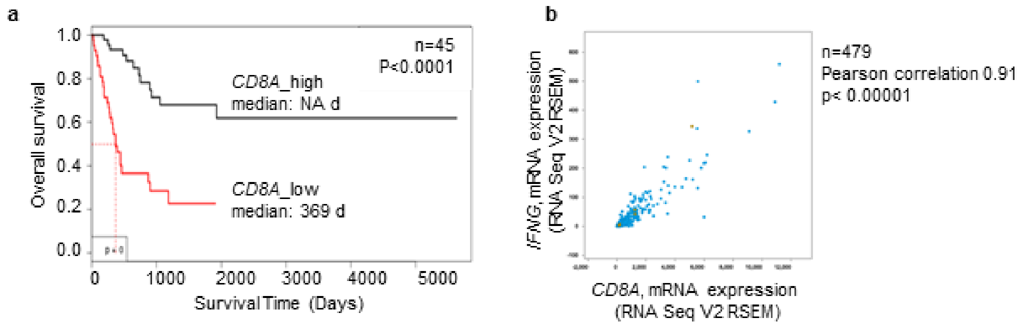

Supplementary Figure 6| *CD8A* mRNA correlates with survival and is co-expressed with *IFNG*. (**a**) Kaplan Meier survival curves for *CD8A* mRNA expressing TCGA melanomas, p-value shown from Log-rank tests. Multivariate p-value corrected for age: p=1.67e-05, n=90, 40 events; (**b**) *CD8A* and *IFNG* mRNA are co-expressed in TCGA samples (yellow points indicate samples mutated in either gene).

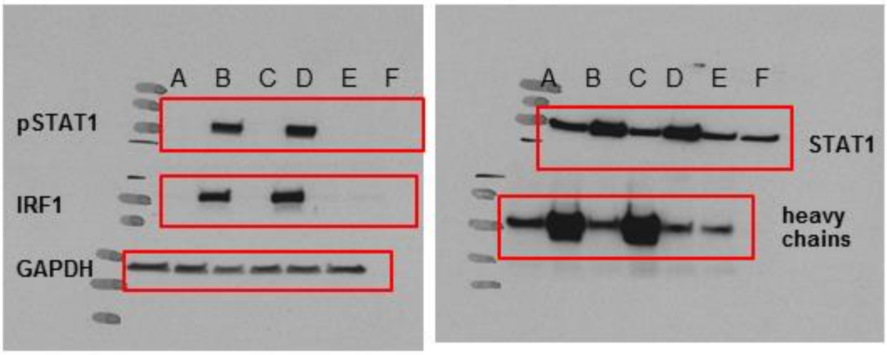

Fig. 1d: A, Ma-Mel-36_bulk
B, Ma-Mel-36_bulk+ IFNγ
C, Ma-Mel-36_sens
D, Ma-Mel-36_sens + IFNγ
E, Ma-Mel-36_res
F, Ma-Mel-36_res + IFNγ

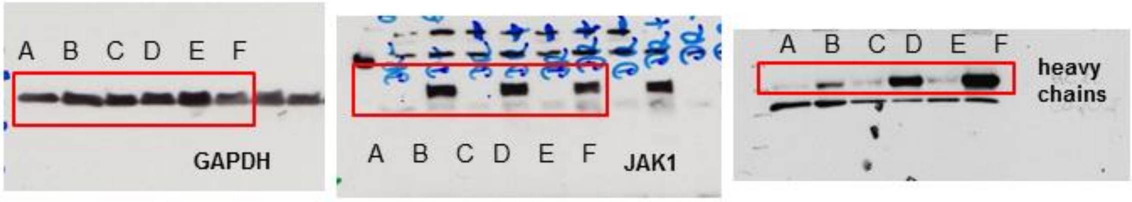

Fig. 1e: A, Ma-Mel-36_res
B, Ma-Mel-36_sens
C, Ma-Mel-36_res + IFNα
D, Ma-Mel-36_sens + IFNγ
E, Ma-Mel-36_res + IFNγ
F, Ma-Mel-36_sens + IFNγ

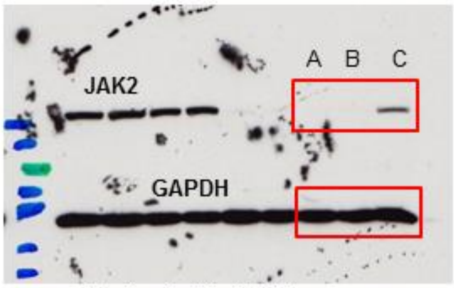

Fig. 2c: A, Ma-Mel-54a
B, Ma-Mel-54b
C, Ma-Mel-61b

Supplementary Figure 7| Full blots of immunoblots shown in the main figures of the manuscript. The respective main figures are indicated.

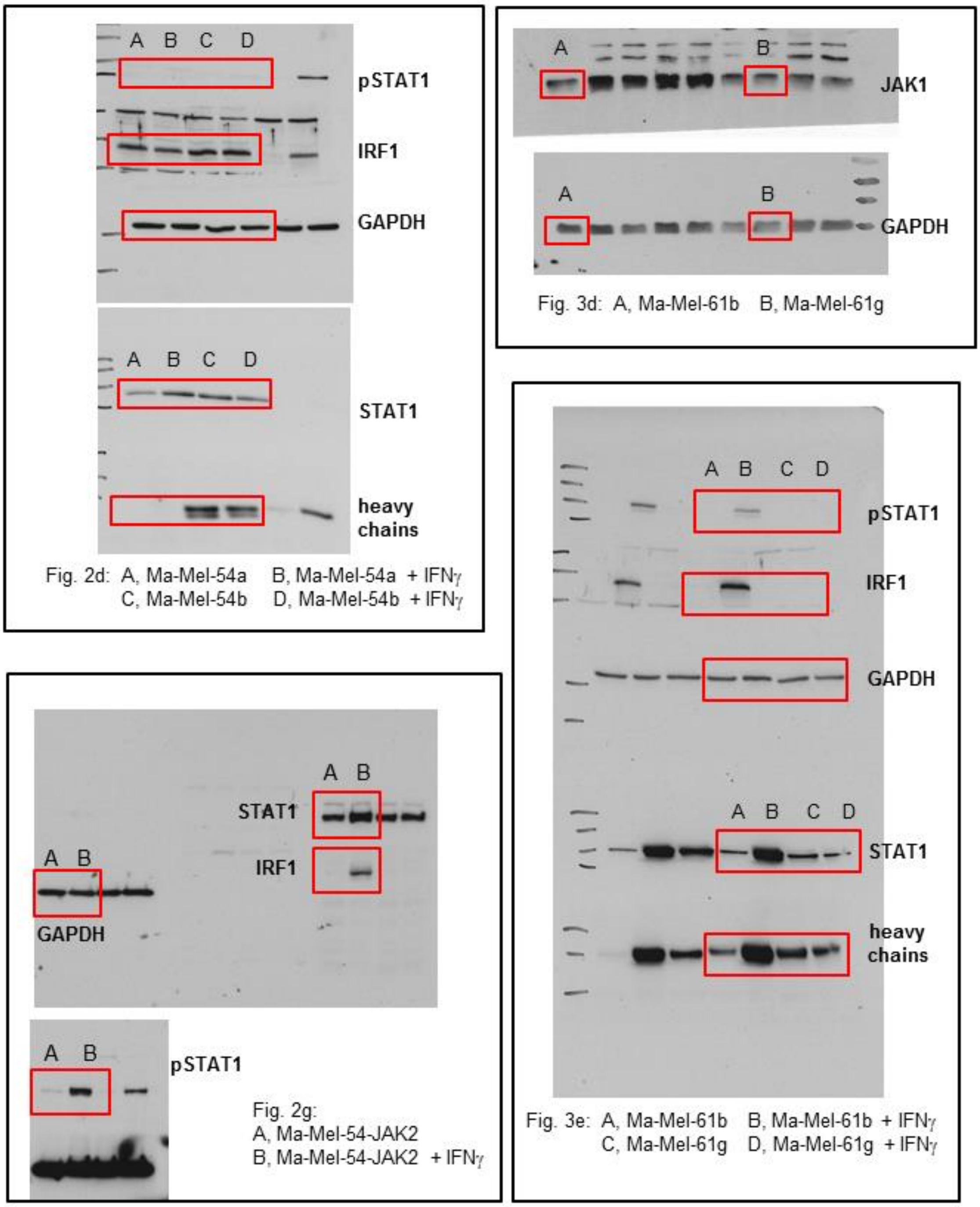

Supplementary Figure 8| Full blots of immunoblots shown in the main figures of the manuscript. The respective main figures are indicated.

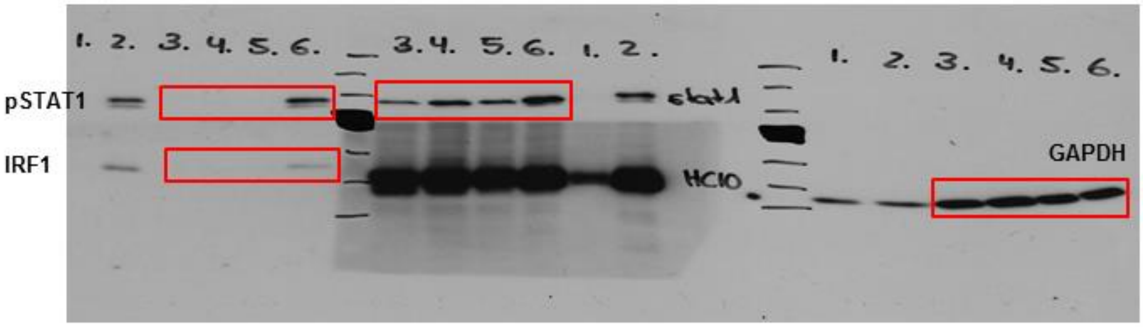

Fig. 3f: 3, Ma-Mel-61g 4, Ma-Mel-61g_JAK1 5, Ma-Mel-61g + IFNγ 6, Ma-Mel-61g_JAK1+ IFNγ

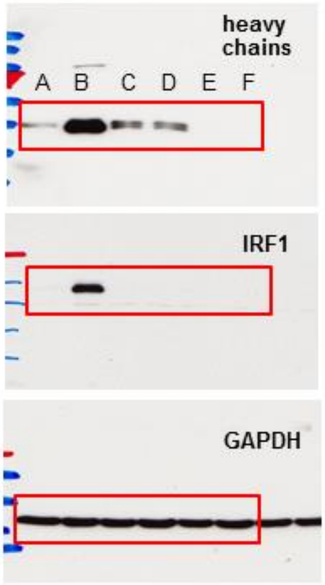

Fig. 4b: A, Ma-Mel-61b
B, Ma-Mel-61b + IFNγ
C, Ma-Mel-61g
D, Ma-Mel-61g + IFNγ
E, Ma-Mel-61h
F, Ma-Mel-61h + IFNγ

Supplementary Figure 9| Full blots of immunoblots shown in the main figures of the manuscript. The respective main figures are indicated

Supplementary Table 1: Treatment response in patient with and without mutations

Best response under anti-PD1 treatment

Clinical response, anti-PD-1 treatment	CR	PR	MR	SD	DC	PD	X-squared	df	p_chi2
Without mutations	1	9	4	7	21	20	NA	NA	NA
With mutations	1	3	0	1	5	6	2.59	4	0.63

Odds ratio for DC vs. PD (p=0.73, OR=1.25, CI=0.32-5.13)

Best response under anti-CTLA-4 treatment

Clinical response, anti-CTLA-4 treatment	CR	PR	SD	DC	PD	X-squared	df	p_chi2
Without mutations	2	12	10	24	67	NA	NA	NA
With mutations	1	2	2	5	9	1.31	3	0.73

Odds ratio for DC vs. PD (p=0.47, OR=0.64, CI=0.2-2.34)

Supplementary Table 2. Genes covered in the applied sequencing panel

Nr.	Gene	Chr.	Location (GRCh37)	Target Bases	Bases covered	Primer pairs	Percentage covered
1	*IRF3*	19	50162835	1,579	1,579	23	100.0
2	*IRF1*	5	131819632	1,158	1,158	21	100.0
3	*STAT2*	12	56737162	3,294	3,294	58	100.0
4	*STAT1*	2	191835418	2,743	2,743	51	100.0
5	*IRF9*	14	24631343	1,342	1,155	19	86.1
6	*IFNAR2*	21	34614217	1,910	1,910	32	100.0
7	*IFNAR1*	21	34697350	1,894	1,798	32	94.9
8	*JAK2*	9	5021977	3,859	3,859	65	100.0
9	*IFNGR1*	6	137519157	1,751	1,646	25	94.0
10	*IFNGR2*	21	34775839	1,231	1,138	21	92.4
11	*JAK1*	1	65300234	3,945	3,945	66	100.0
12	*TYK2*	19	10461499	4,024	3,761	58	93.5
